# The AHR represses nucleotide excision repair and apoptosis and contributes to UV-induced skin carcinogenesis

**DOI:** 10.1038/s41418-018-0160-1

**Published:** 2018-07-16

**Authors:** Marius Pollet, Siraz Shaik, Melina Mescher, Katrin Frauenstein, Julia Tigges, Stephan A. Braun, Kevin Sondenheimer, Mana Kaveh, Anika Bruhs, Stephan Meller, Bernhard Homey, Agatha Schwarz, Charlotte Esser, Thierry Douki, Christoph F. A. Vogel, Jean Krutmann, Thomas Haarmann-Stemmann

**Affiliations:** 1 IUF—Leibniz-Research Institute for Environmental Medicine, 40225 Düsseldorf, Germany; 2 Department of Dermatology, Medical Faculty, Heinrich-Heine University, 40225 Düsseldorf, Germany; 3 Department of Dermatology and Allergology, Kiel University, 24105 Kiel, Germany; 4 University Grenoble Alpes, INAC, CNRS, SyMMES UMR 3819, F-38000 Grenoble, France; 5 Department of Environmental Toxicology and Center for Health and the Environment, University of California, Davis, CA 95616 USA

**Keywords:** Cancer, Cell biology

## Abstract

Ultraviolet B (UVB) radiation induces mutagenic DNA photoproducts, in particular cyclobutane pyrimidine dimers (CPDs), in epidermal keratinocytes (KC). To prevent skin carcinogenesis, these DNA photoproducts must be removed by nucleotide excision repair (NER) or apoptosis. Here we report that the UVB-sensitive transcription factor aryl hydrocarbon receptor (AHR) attenuates the clearance of UVB-induced CPDs in human HaCaT KC and skin from SKH-1 hairless mice. Subsequent RNA interference and inhibitor studies in KC revealed that AHR specifically suppresses global genome but not transcription-coupled NER. In further experiments, we found that the accelerated repair of CPDs in AHR-compromised KC depended on a modulation of the p27 tumor suppressor protein. Accordingly, p27 protein levels were increased in AHR-silenced KC and skin biopsies from AHR^−/−^ mice, and critical for the improvement of NER. Besides increasing NER activity, AHR inhibition was accompanied by an enhanced occurrence of DNA double-strand breaks triggering KC apoptosis at later time points after irradiation. The UVB-activated AHR thus acts as a negative regulator of both early defense systems against carcinogenesis, NER and apoptosis, implying that it exhibits tumorigenic functions in UVB-exposed skin. In fact, AHR^−/−^ mice developed 50% less UVB-induced cutaneous squamous cell carcinomas in a chronic photocarcinogenesis study than their AHR^+/+^ littermates. Taken together, our data reveal that AHR influences DNA damage-dependent responses in UVB-irradiated KC and critically contributes to skin photocarcinogenesis in mice.

## Introduction

Exposure to ultraviolet B (UVB) radiation is the major risk factor for cutaneous squamous cell carcinoma (SCC), one of the most frequent malignancies in humans [1, 2]. When skin is exposed to solar radiation, high-energy UVB rays penetrate into the epidermis where they are mainly absorbed by the DNA of keratinocytes (KC). The resulting DNA photoproducts, in particular cyclobutane pyrimidine dimers (CPDs), are highly mutagenic and critically involved in the development of SCC [1, 2]. Depending on the extent of DNA damage, KC either initiate nucleotide excision repair (NER) or apoptosis to preserve genomic integrity [3, 4]. Failure of these early defense mechanisms may give rise to initiated cells, whose spread is further promoted by UVB-activated signaling pathways facilitating proliferation, apoptosis resistance and impairment of host immune responses [1, 2].

NER is divided into two distinct sub-pathways: transcription-coupled repair (TCR), which quickly removes DNA adducts in actively transcribed genes, and global genome repair (GGR), which removes DNA lesions throughout the entire genome [3]. Both subpathways only differ in their way of DNA damage recognition. In case of TCR, Cockayne Syndrome (CS)-A and CSB proteins recognize the stalled RNA polymerase II and serve as damage sensors. In GGR, a complex consisting of XPC, RAD23B, and centrin-2 is responsible for DNA damage recognition. Upon damage verification by XPA, DNA is unwinded by the helicases XPB and XPD, two components of the general transcription factor TFIIH. Subsequently, the damage-containing DNA fragment is excised by the endonucleases XPF-ERCC1 and XPG and the remaining gap is filled by DNA polymerases. The pivotal role of NER in skin photocarcinogenesis is illustrated by the autosomal recessive NER disorder *Xeroderma Pigmentosum* (XP). XP patients with a deficiency in GGR have a dramatically increased risk of developing SCC and other skin cancers [5]. In contrast to the repair process, the precise regulation of NER in response to UVB radiation is still not completely understood.

The aryl hydrocarbon receptor (AHR) is a ligand-activated transcription factor that mediates the toxic effects of dioxins, polycyclic aromatic hydrocarbons and related environmental chemicals [6, 7]. In its inactive state, AHR rests in a cytosolic multiprotein complex. Upon ligand-binding, AHR shuttles in the nucleus, dimerizes with AHR nuclear translocator and binds to xenobiotic-responsive elements in the enhancer of target genes to initiate their transcription. AHR target genes encode drug-metabolizing enzymes, such as cytochrome P450 1A1, as well as proteins controlling cell division, differentiation and apoptosis [6, 7]. Another target is the AHR repressor (AHRR), a negative feedback regulator, which, depending on cell-type and tissue, may compete with AHR for both AHR nuclear translocator- and xenobiotic-responsive element-binding [8]. Beside the canonical AHR pathway, AHR activation often affects other signal transduction pathways, including NF-κB and EGFR signaling [6, 7].

AHR is expressed in all cutaneous cell-types and contributes to physiological as well as pathophysiological processes [9]. In epidermal KC, AHR activation results from the absorbance of UVB rays by tryptophan and the subsequent generation of 6-formylindolo [3,2-*b*] carbazole [10]. This tryptophan photoproduct is a high-affinity ligand for AHR [11] and, among others, induces the expression of cytochrome P450 1A1 and cyclooxygenase-2 [9, 10]. As these enzymes play critical roles in tumor initiation and promotion, it was proposed that the UVB-activated AHR contributes to photocarcinogenesis [12]. This idea is supported by our recent observations that AHR triggers immunosuppression [13] and anti-apoptosis in response to UVB exposure [14]. Interestingly, independent reports revealed that AHR-antagonizing polyphenols, such as epigallocatechin-3-gallate [15], stimulate the repair of UVB-induced CPDs [16], suggesting that AHR signaling may influence NER.

In the present study, we directly tested this hypothesis by assessing the impact of AHR on the removal of UVB-induced CPDs in human HaCaT KC and SKH-1 hairless mice. We found that AHR inhibition enhances the removal of CPDs in vitro and in vivo. Mechanistic studies revealed that AHR specifically dampens GGR activity by decreasing the protein level of the tumor suppressor p27^KIP1^ (p27), which probably affects DNA repair independently from its main function, i.e., induction of cell-cycle arrest. In addition to its positive effect on NER, AHR inhibition enhances the apoptotic clearance of remaining CPD-positive KC at later time points and thus enforces both early defense mechanisms against photocarcinogenesis. Accordingly, chronic UVB irradiation experiments conducted in AHR^+/+^ and AHR^−/−^ SKH-1 mice revealed a dramatically reduced SCC formation in the AHR^−/−^ animals.

## Results

### AHR inhibition accelerates the removal of UVB-induced CPDs in human KC and murine skin

**Fig. 1:**
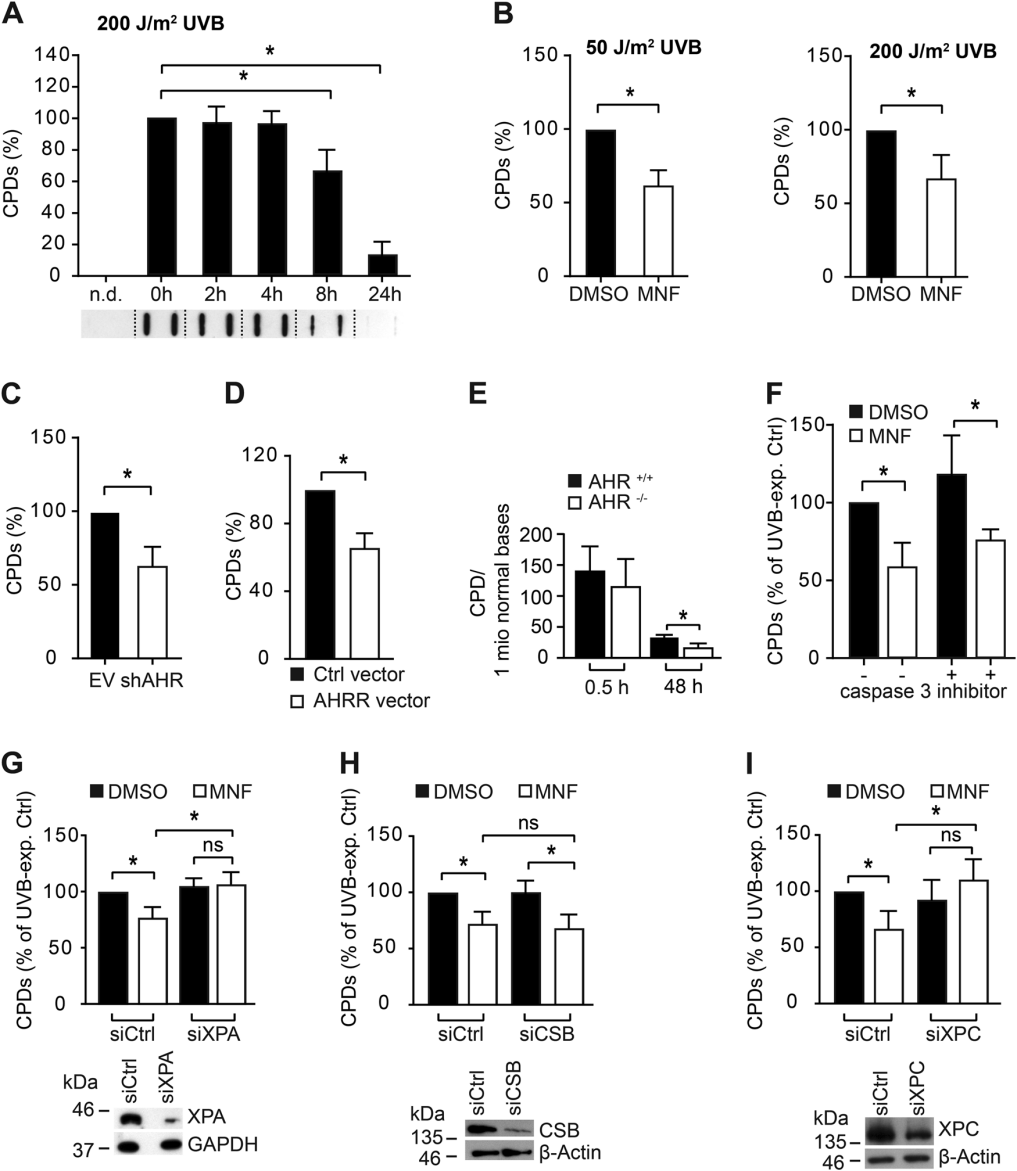
Chemical and genetic inhibition of AHR enhances the removal of UVB-induced CPDs by modulating GGR. **a** Time-dependent clearance of CPDs in HaCaT KC irradiated with 200 J/m^2^ UVB. Cells were harvested at 0 (directly), 2, 4, 8, and 24 h after irradiation. Below the diagram, a representative SWB result is shown. **b** HaCaT KC were irradiated with 50 J/m^2^ (left panel) and 200 J/m^2^ (right panel) UVB and immediately treated with 0.1% DMSO or 20 µM MNF. After 4 h, CPD content was determined. **c** HaCaT-EV and HaCaT-shAHR KC were exposed to 200 J/m^2^ UVB and 4 h later the CPD content was measured. **d** HaCaT KC were transfected with Ctrl. vector or an AHRR overexpression vector and 24 h later irradiated with 200 J/m^2^ UVB. After 4 h, the amount of CPDs was determined. **e** HPLC-MS/MS-based analysis of CPDs in the DNA of skin samples from AHR^+/+^ and AHR^-/-^ SKH-1 mice 0.5 h and 48 h after exposure to a single dose of 185 mJ/cm^2^ UVB. **f** HaCaT KC were exposed to 200 J/m^2^ and treated with MNF (20 µM) and caspase inhibitor Ac-DEVD-CHO (20 µM) alone or in combination. After 4 h, CPD content was measured. To assess if AHR affects TCR and/or GGR, we transiently silenced the expression of XPA (**g**), CSB (**h**) and XPC (**i**) and 24 h later irradiated the cells with 200 J/m^2^ UVB. Subsequently, the KC were treated for 4 h with MNF (20 µM) or 0.1% DMSO and CPD content was analyzed. Measurement of CPD content was carried out by SWB. For each experiment, the CPD content of the respective UVB-exposed controls was set to 100%. ns not significant. *p ≤ 0.05

To assess if AHR affects the removal of CPDs, we irradiated human HaCaT KC with 200 J/m^2^ UVB and monitored CPD clearance by southwestern slot blot (SWB) analysis over time. CPD content stayed elevated in irradiated cells for the first 4 h after irradiation and then gradually declined to only 10% after 24 h (Fig. 1a). As expected (and true for all of the following CPD SWB analyses), we did not detect a CPD signal in sham-exposed KC. Next, we exposed HaCaT KC to 50 J/m^2^ and 200 J/m^2^ UVB (approximately equivalent to 0.25 and 1 minimal erythema dose for a fair-skinned individual [17]) and subsequently treated the cells with the AHR antagonist 3′-methoxy-4′-nitroflavone (MNF, 20 µM) to exclude putative UV-filtering effects. 4 h after irradiation, the CPD content in MNF-treated cells was 37 and 33% lower than in solvent controls (Fig. 1b). Similar results were obtained when primary human epidermal KC were treated with MNF (data not shown). In stable AHR-knockdown cells (HaCaT-shAHR), the amount of UVB-induced CPDs was reduced by 37% 4 h after irradiation, as compared to irradiated empty vector control cells (HaCaT-EV) (Fig. 1c), thus excluding putative off-target effects of MNF. In addition, ectopic overexpression of AHR’s feedback inhibitor AHRR accelerated the removal of UVB-induced CPDs (Fig. 1d). To confirm an influence of AHR on CPD removal in vivo, AHR^+/+^ and AHR^−/−^ SKH-1 hairless mice were irradiated once with 185 mJ/cm^2^ UVB and 0.5 h and 48 h thereafter skin biopsies were taken. CPD content was quantified in complete biopsies by HPLC-MS/MS. In the skin of both genotypes, the distribution between TT, TC and CT CPDs was approximately 55:35:10 (Supplementary Figure S1), which is in accordance with previously published results [18]. Immediately after UVB exposure, CPD content did not significantly differ between AHR genotypes. However, after 48 h approximately 50% less CPDs were present in the skin of AHR^−/−^ mice as compared to AHR^+/+^ mice (Fig. 1e), thus demonstrating that the negative effect of AHR on CPD clearance is also present in vivo.

### AHR regulates NER

In all of the in vitro experiments, CPD content was determined in adherent KC. Also, co-exposure of MNF-treated HaCaT KC to Ac-DEVD-CHO (20 µM), an inhibitor of effector caspases, did not affect the MNF-mediated acceleration of CPD removal (Fig. 1f). These results indicate that the reduced CPD levels observed in AHR-compromised UVB-irradiated human KC were not due to an early apoptotic demise of damaged cells, but likely resulted from altered NER. To assess whether acceleration of CPD removal induced by AHR inhibition was NER-dependent, we silenced the expression of XPA. In contrast to control cells, MNF exposure of UVB-exposed XPA-silenced KC failed to affect CPD removal (Fig. 1g), strongly indicating that AHR modulates NER activity. We next investigated whether AHR antagonism affects GGR and/or TCR. Therefore, we transiently transfected HaCaT KC with siRNAs targeted against XPC and CSB. After 24 h, transfected cells were irradiated with 200 J/m^2^ UVB and treated with 20 µM MNF or solvent. We found that MNF significantly increased CPD clearance in CSB-knockdown KC (Fig. 1h), but not in XPC-silenced KC (Fig. 1i). These results provide evidence that AHR inhibition accelerates CPD removal by specifically modulating GGR.

### p27 is causally involved in the AHR-dependent inhibition of NER

**Fig. 2:**
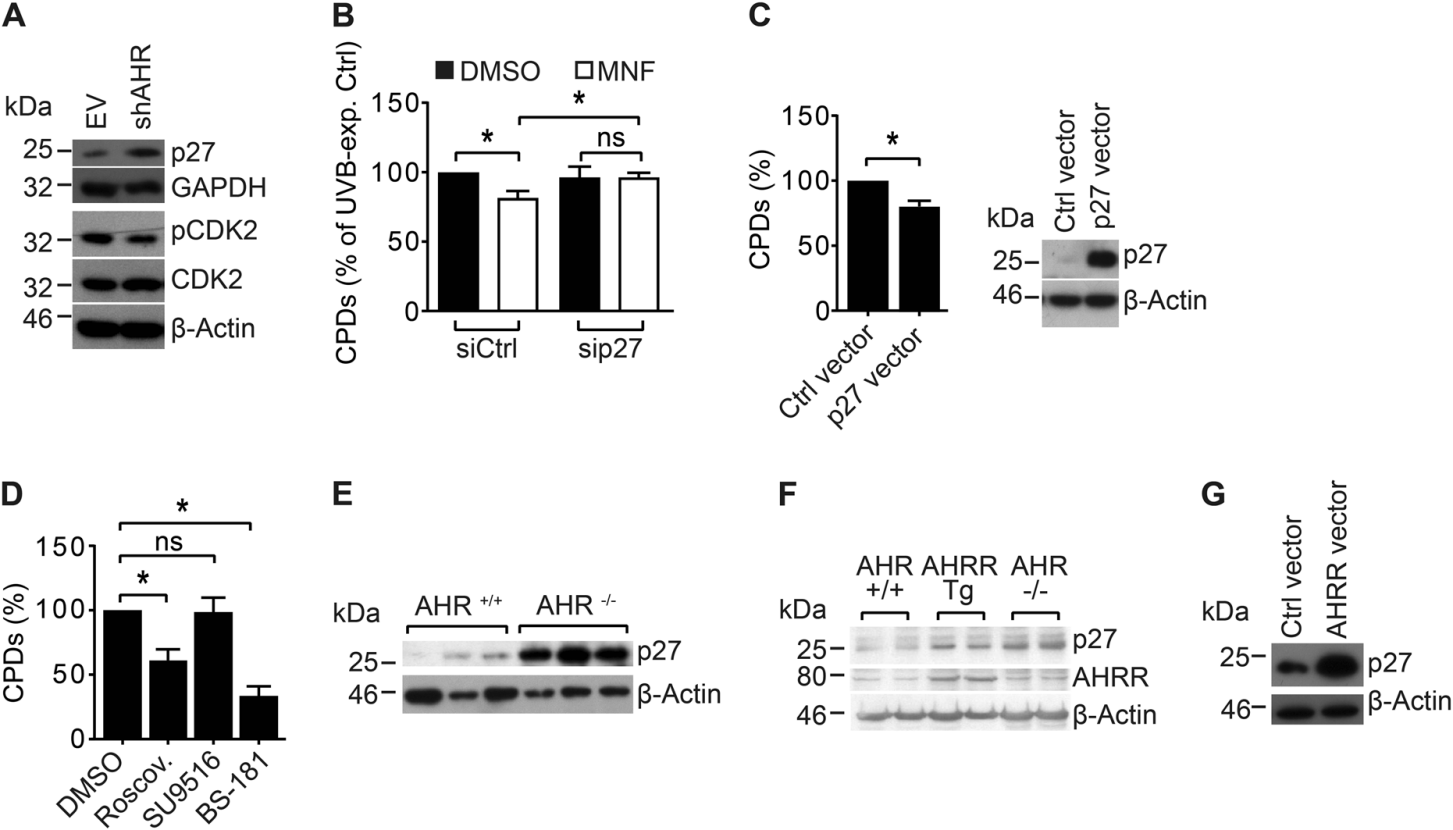
AHR inhibits GGR by modulating the protein level of the tumor suppressor p27. **a** Western blot analysis of p27, pCDK2, and CDK2 in untreated HaCaT-EV and HaCaT-shAHR KC (representative blots). **b** HaCaT KC were transiently transfected with *p27*-targeted siRNA and Ctrl. siRNA. After 24 h, the cells were irradiated with 200 J/m^2^ UVB and treated with 20 µM MNF or 0.1% DMSO. After 4 h, the CPD content was compared by SWB. **c** HaCaT KC were transiently transfected with Ctrl. vector or a p27 expression plasmid. After 24 h, the cells were exposed to 200 J/m^2^ UVB and 4 h later the CPD content was determined. **d** HaCaT KC were irradiated with 200 J/m^2^ UVB and subsequently treated with 1 µM roscovitine, 500 nM SU9516, 125 nM BS-181 or 0.1% DMSO. After 4 h, the CPD content was analyzed by SWB. **e** Protein lysates from skin samples of AHR^+/+^ and AHR^−/−^ SKH-1 mice were analyzed for p27 protein content by SDS-PAGE/western blotting. **f** Protein lysates from skin samples of AHR^+/+^, AHRR Tg and AHR^−/−^ B6 mice were analyzed for p27 and AHRR protein content by SDS-PAGE/western blotting. **g** HaCaT KC were transiently transfected with an overexpression plasmid for rat AHRR or empty vector. After 24 h, the p27 protein level was compared by SDS-PAGE/western blot analysis (representative blot). ns not significant. *p ≤ 0.05

We have previously reported that AHR-compromised KC contain an increased level of the CDK inhibitor p27 [14]. Accordingly, the p27 protein level was strongly elevated in HaCaT-shAHR KC as compared to HaCaT-EV KC, and associated with a reduced phosphorylation of CDK2 (T-160) (Fig. 2a). As enhanced p27 levels were reported to correlate with DNA repair capacity in human peripheral blood lymphocytes [19], we assessed whether elevated p27 levels may be causative for the acceleration of NER. We silenced p27 expression using transient RNAi and investigated if MNF exposure still accelerates NER. We found that in contrast to respective control cells, knockdown of p27 abolished the MNF-mediated increase in CPD removal (Fig. 2b). In addition, transient overexpression of p27 in HaCaT KC resulted in an enhanced clearance of UVB-induced CPDs (Fig. 2c), demonstrating that p27 can per se stimulate NER activity.

### p27 enhances GGR activity independently from cell-cycle arrest

Interestingly, FACS analyses of UVB-exposed HaCaT KC treated with DMSO or MNF did not show any significant differences in the cell-cycle profiles, at least within the first 4 h after irradiation (supplementary figure S2), suggesting that p27 affects GGR independently from CDK2/CDK4 inhibition. To elucidate if p27 alters GGR activity in a CDK-dependent manner, we tested the potential of various chemical CDK inhibitors on their ability to alter CPD removal in HaCaT KC. In fact, treatment of UVB-exposed KC with roscovitine, an inhibitor of CDK1, CDK2, CDK5, and CDK7, resulted in an enhanced removal of CPDs 4 h after UVB irradiation (Fig. 2d). In contrast, exposure to SU9516, an inhibitor of CDK1, CDK2, and CDK4, had no effect on the cellular amount of UVB-induced CPDs (Fig. 2d), again supporting our notion that p27 alters GGR activity independently from CDK2 and CDK4. Interestingly, treatment of UVB-irradiated cells with BS-181, a specific inhibitor of CDK7, mimicked the positive effect of p27 overexpression on CPD repair (Fig. 2d). Although CDK7 is known to be involved in the regulation of NER [20], subsequent co-immunoprecipitation analyses in sham- and UVB-exposed HaCaT KC did not reveal a direct protein–protein interaction of p27 and CDK7 (data not shown). Thus, p27 may either affect GGR independently from CDK7 or through an indirect modulation of CDK7 function.

### AHR regulates the proteasomal degradation of p27

**Fig. 3:**
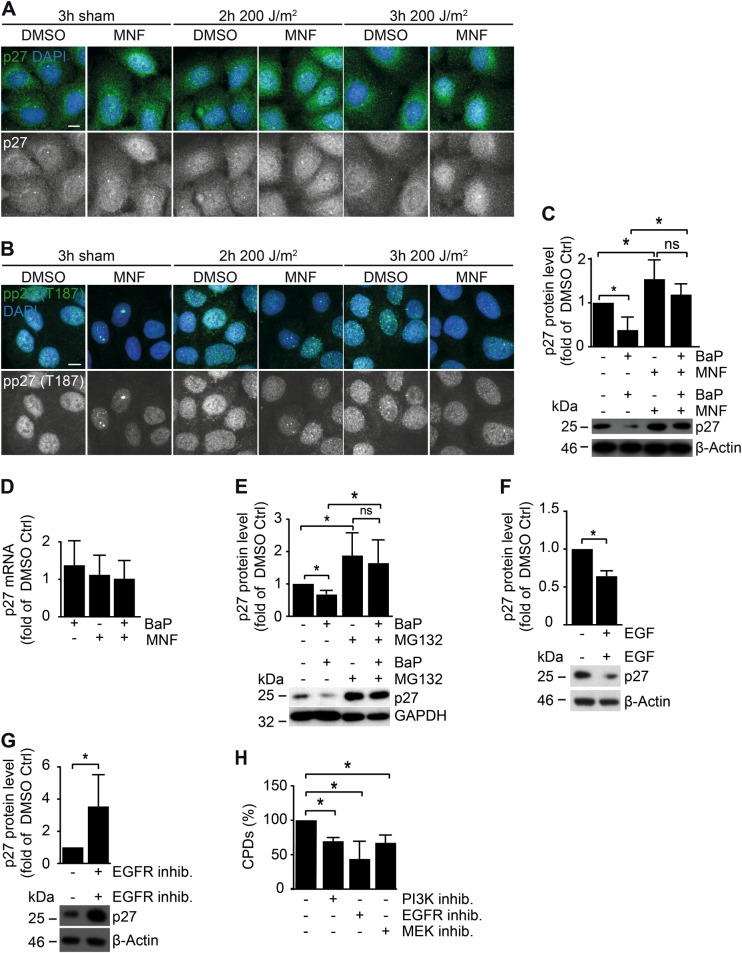
AHR-dependent modulation of the p27 protein level. HaCaT KC were irradiated with 0 and 200 J/m^2^ UVB and treated with 20 µM MNF or 0.1% DMSO. After 2 h and 3 h, cells were fixed and antibody stained for **a** p27 and **b** pp27 T-187 (scale: 10 µm). **c** HaCaT KC were treated with 2.5 µM BaP and 20 µM MNF alone or in combination. Control cells were treated with 0.2% DMSO. Protein content of p27 was assessed by SDS-PAGE/western blot analysis (right). **d** HaCaT cells were exposed to 2.5 µM BaP, 20 µM MNF or both. After 24 h, copy numbers of *p27* were determined by qPCR. Data are shown as fold of DMSO ctrl. **e** HaCaT KC were treated with 2.5 µM BaP, 10 µM MG132 and 0.2% DMSO alone or in combination. Protein content of p27 was assessed by SDS-PAGE/western blotting (top: quantification, bottom: representative WB). **f** HaCaT-shAHR KC were treated for 2 h with 50 ng/ml EGF or solvent. Subsequently, p27 protein level was determined by SDS-PAGE/western blot analysis (top: quantification, bottom: representative WB). **g** HaCaT-EV KC were treated for 2 h with 10 µM of the EGFR inhibitor PD153035 or 0.1% DMSO. Subsequently, p27 protein level was determined by SDS-PAGE/western blotting (top: quantification, bottom: representative WB). **h** HaCaT KC were irradiated with 200 J/m^2^ UVB and immediately treated with 0.1% DMSO, 10 µM PD153035 (EGFR inhibitor), 1 µM Wortmannin (PI3K inhibitor) or 10 µM PD98059 (MEK inhibitor). After 4 h, CPD content was determined by SWB. *p ≤ 0.05

To assess the in vivo relevance of the observed AHR-dependent effects on p27 level, we determined the p27 protein content in the skin of two strains of AHR^+/+^ and AHR^−/−^ mice. As expected, protein levels of p27 were higher in the skin of AHR^−/−^ SKH-1 mice (Fig. 2e) and AHR^−/−^ B6 mice (Fig. 2f) as compared to littermate controls. Also, overexpression of AHRR increased p27 protein content in the skin of transgenic B6 (AHRR Tg) mice [21] (Fig. 2f) as well as in transiently transfected HaCaT KC (Fig. 2g). We next tested whether transcriptional or post-transcriptional events were responsible for these effects. As expected, immunofluorescence (IF) staining of HaCaT KC revealed a nuclear accumulation of AHR in response to UVB irradiation, which was attenuated by MNF treatment (Supplementary Figure S3A). Exposure of HaCaT KC to MNF alone resulted in an increase of nuclear p27 (Fig. 3a). In comparison to irradiated HaCaT KC, an increased amount of nuclear p27 was also present in MNF-treated HaCaT KC 2 h and 3 h after exposure to 200 J/m^2^ UVB (Fig. 3a). The increased levels of p27 were accompanied by a reduced phosphorylation of CDK2 (Supplementary Figure S3B). The differences in nuclear p27 were reflected by IF stainings using a phospho-p27 (T-187) antibody (Fig. 3b). The amino acid T-187 serves as substrate for CDK2 and targets p27 to proteolysis [22]. Further mechanistic studies in HaCaT cells using chemical AHR ligands confirmed that AHR activity affected p27 on the protein level. Treatment of HaCaT KC with the AHR agonist benzo[*a*]pyrene (BaP) reduced the p27 protein level, whereas exposure to MNF increased it (Fig. 3c). These changes were not retrievable on the mRNA level (Fig. 3d). Accordingly, the BaP-induced reduction of p27 protein was abrogated by co-treatment with the proteasome inhibitor MG-132 (Fig. 3e). Interestingly, exposure of HaCaT-shAHR KC to EGF attenuated the elevated p27 protein level (Fig. 3f), whereas treatment of HaCaT-EV KC with the EGFR inhibitor PD153035 was sufficient to increase it (Fig. 3g). Importantly, treatment of HaCaT KC with inhibitors for EGFR (PD153035) and its effector pathways PI3K/AKT (Wortmannin) and MEK/ERK (PD98059) accelerated the CPD removal 4 h after UVB irradiation (Fig. 3h). These results are consistent with the assumption that AHR attenuates GGR in UVB-irradiated KC by promoting proteolysis of p27, most likely involving EGFR and downstream signal transduction.

### AHR inhibition increases UVB-induced apoptosis in KC by enhancing DNA double strand break formation

**Fig. 4:**
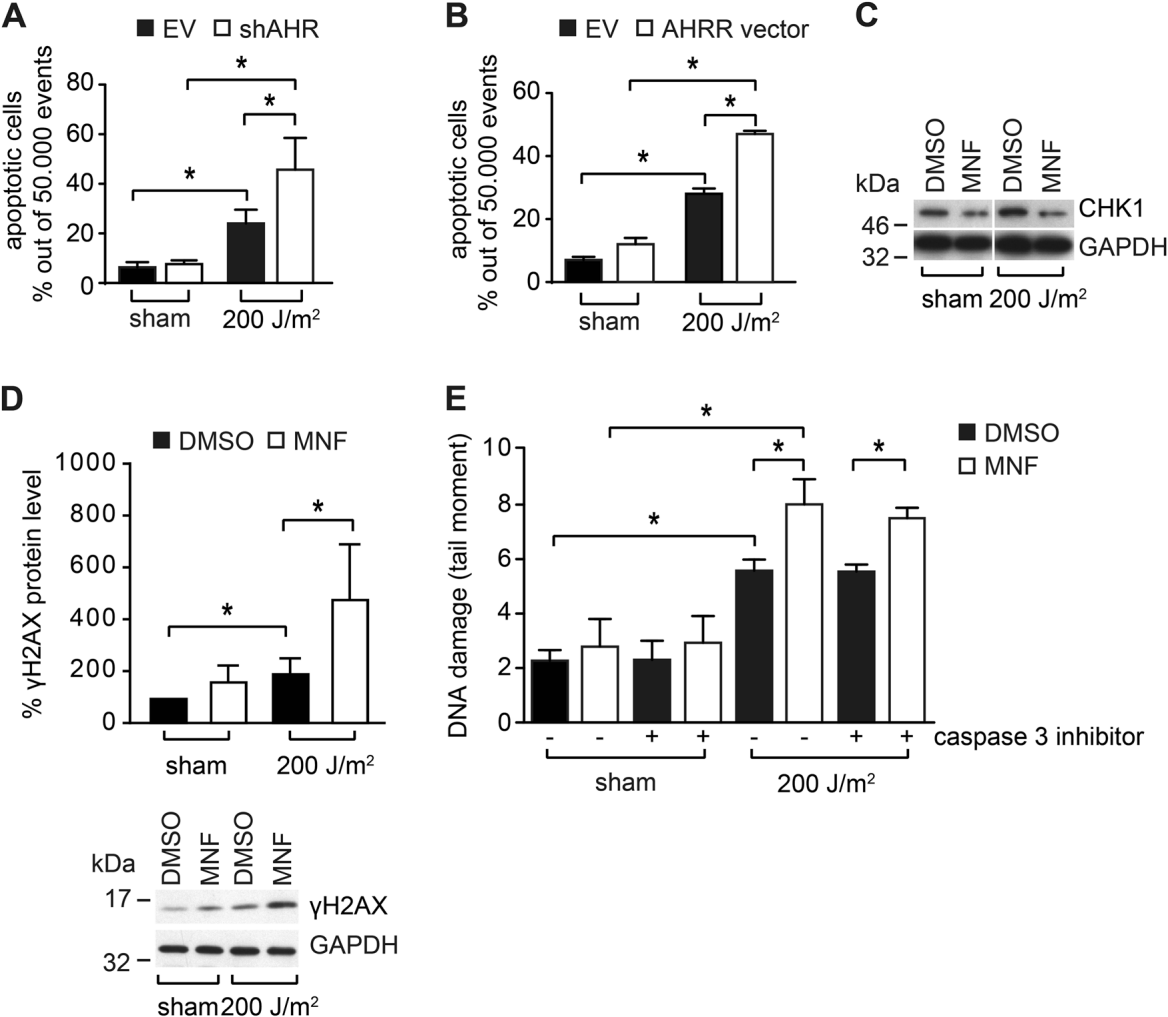
AHR inhibition increases UVB-induced apoptosis and is associated with DSB formation. **a** HaCaT-EV and HaCaT-shAHR KC were irradiated with 0 and 200 J/m^2^ UVB. After 24 h, the amount of dead cells was analyzed by Annexin V/PI staining. **b** HaCaT KC were transiently transfected with an expression vector for rat AHRR or empty vector. After 24 h, the KC were exposed to 200 J/m^2^ UVB and another 24 h later, the amount of dead cells was determined by Annexin V/PI staining. **c** Western blot analysis of CHK1 in HaCaT KC 24 h after irradiation with 0 and 200 J/m^2^ UVB (representative blot). **d** HaCaT KC were irradiated with 0 and 200 J/m^2^ UVB. After 18 h, γH2AX levels were assessed by SDS-PAGE/western blotting. **e** HaCaT KC were irradiated with 0 and 200 J/m^2^ UVB and immediately treated with MNF (20 µM) and DMSO alone or in combination with the caspase inhibitor Ac-DEVD-CHO (20 µM). After 18 h, DSBs were detected by neutral comet assay analyses. *p ≤ 0.05

An increase in NER should partially reduce UVB-induced KC apoptosis [23]. This is in contrast to our previous report that AHR inhibition enhances UVB-induced KC apoptosis [14]. Accordingly, FACS-based analyses of Annexin V/PI-stained cells revealed a stronger apoptotic response in HaCaT-shAHR KC exposed to 200 J/m^2^ UVB as compared to HaCaT-EV cells (Fig. 4a, Supplementary Figure S4A). The AHR-dependent difference in UVB-induced apoptosis was confirmed on the level of caspase-3 activity (Supplementary Figure S4B). Also, UVB irradiation of AHRR-overexpressing HaCaT KC resulted in a more pronounced apoptotic response, as compared to control cells (Fig. 4b, Supplementary Figure S4C). In our previous study we showed that the anti-apoptotic action of AHR is due to the loss of checkpoint kinase-1 (CHK1) expression [14]. CHK1 is a stress kinase that is directly activated in response to DNA damage to prevent cell-cycle progression [24]. In fact, exposure of HaCaT KC to 20 µM MNF resulted in a reduced expression of CHK1 protein, both in sham and UVB-irradiated cells (Fig. 4c). As CHK1 is also required for homologous recombination repair (HRR) [25], we next asked if DNA double-strand breaks (DSBs) may be responsible for the enhancement of apoptosis observed in AHR-compromised KC. Accordingly, phosphorylation of histone H2AX (γH2AX), an established marker for DSBs, was significantly stronger in UVB-exposed MNF-treated HaCaT as compared to irradiated control KC (Fig. 4d). Moreover, neutral comet assay analyses demonstrated an enhanced occurrence of DSBs in MNF-treated HaCaT KC 18 h after UVB irradiation (Fig. 4e). The DSBs still occurred when the cells were co-exposed to a caspase inhibitor, indicating that they were not produced by apoptosis-related DNases (Fig. 4e). These results indicate that the enhanced UVB-induced apoptosis in AHR-compromised cells is driven by an increased formation of DSBs and that it occurs independently of NER, thus reconciling our seemingly contradictory observations on NER and apoptosis in AHR-deficient KC.

### Reduced photocarcinogenesis in AHR^−/−^ mice

**Fig. 5:**
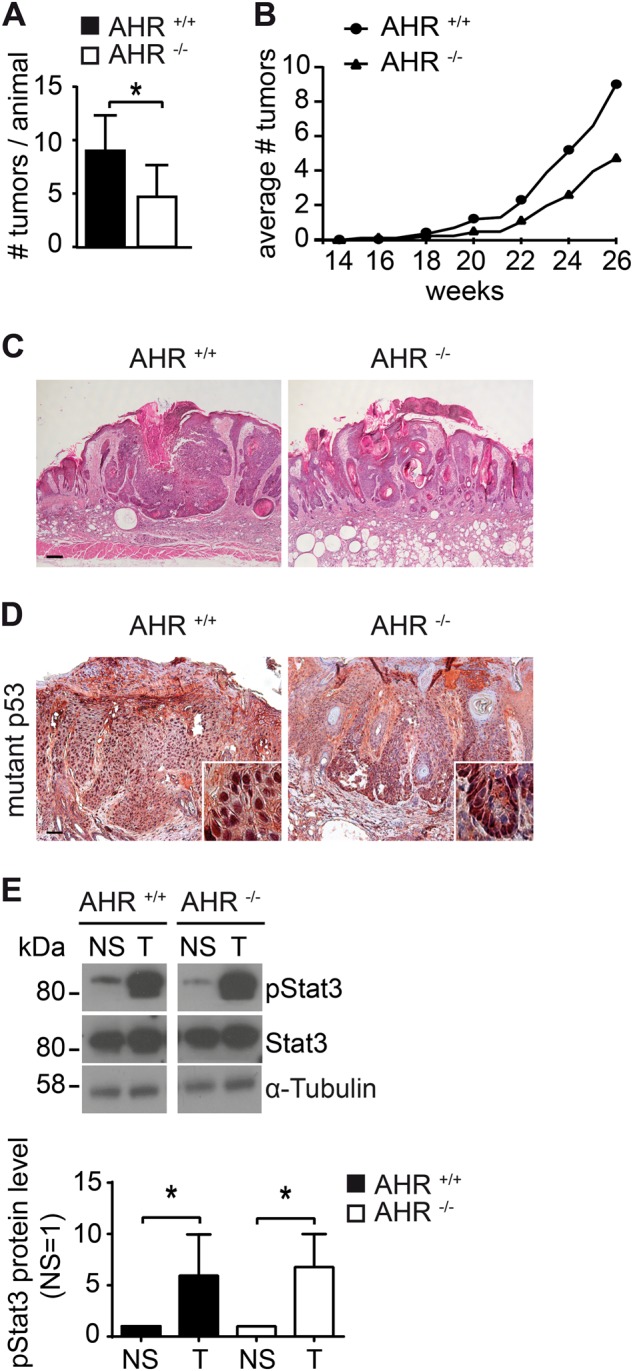
AHR contributes to UVB-induced skin carcinogenesis in SKH-1 hairless mice. **a** Ten AHR^+/+^ and eight AHR^−/−^ SKH-1 mice were chronically exposed to UVB radiation as described in Materials and Methods section. 2 weeks after the last irradiation, total numbers of SCCs were compared. **b** Time-course of UVB-induced SCC development in the skin of AHR^+/+^ and AHR^−/−^ SKH-1 mice. **c** Representative histological pictures from primary SCCs from AHR^+/+^ and AHR^−/−^ mice (scale: 200 µm). *p ≤ 0.05. **d** Immunohistochemical staining of lesional and adjacent non-lesional skin of AHR^+/+^ and AHR^−/−^ SKH-1 mice with an antibody detecting mutant p53 protein (scale: 300 µm). **e** Protein lysates from lesional (T) and irradiated non-lesional (NS) skin of AHR^+/+^ and AHR^−/−^ SKH-1 mice were analyzed for phosphorylation (at Y-705) and expression of STAT3 by SDS-PAGE/western blotting. *p ≤ 0.05

Our results demonstrate that the UVB-activated AHR is a negative regulator of GGR and apoptosis. We therefore speculated that AHR-deficiency would protect mice against the UVB-induced development of SCC. To test this hypothesis, we conducted a chronic photocarcinogenesis study in AHR^+/+^ and AHR^−/−^ SKH-1 mice. As shown in Fig. 5a, AHR^−/−^ mice developed approximately 50% less skin tumors than their AHR^+/+^ littermates. The animals started to develop skin tumors after 15–17 weeks of UVB exposure (Fig. 5b). There were no genotype-dependent differences in tumor histology (Fig. 5c). As previously reported for hairless mice [26] all UVB-induced skin tumors were SCC ranging from well to poorly differentiated in situ carcinomas to deeply infiltrating tumors. This was confirmed by immunohistochemical analyses revealing an accumulation of mutant p53 protein, a hallmark of UVB-induced SCCs [27, 28], in lesional but not adjacent non-lesional skin from mice of both AHR genotypes (Fig. 5d). In addition, immunoblot analyses exhibited an elevated activation of STAT3 (phosphorylation at Y-705), an established key driver of UVB-induced SCC development [29], in tumor samples from both, AHR^+/+^ and AHR^−/−^ mice, as compared to irradiated non-lesional skin (Fig. 5e). Notably, sham-exposed control animals of both genotypes did not develop any skin tumors (data not shown). Taken together, these data reveal a crucial role of AHR in UVB-induced skin carcinogenesis.

## Discussion

The major findings of the present study are that AHR attenuates the clearance of UVB-induced CPDs by specifically repressing GGR in a p27-dependent manner, and that AHR-deficiency largely protects mice against UVB-induced skin carcinogenesis.

CPDs are primarily responsible for the onset of UVB-induced skin carcinogenesis [28] and their forced repair has been demonstrated to efficiently reduce the incidence of skin cancer in mice [30] and humans [31]. We therefore believe that the reduced SCC development in AHR^−/−^ mice is, at least to a major extent, the consequence of elevated NER activity. This assumption is further supported by the fact that AHR inhibition specifically increased GGR, which is the pivotal DNA repair system restraining photocarcinogenesis. In fact, XP patients suffering from GGR-deficiency have a greatly increased risk of developing skin cancer [5]. In contrast, TCR-deficiency (CS) is not associated with an increased incidence of skin cancer, which is probably due to an enhanced cytotoxicity (but not mutagenicity) in response to UV exposure [32]. Since CPDs are the major trigger for UVB-induced immunosuppression [33], their accelerated repair in AHR^−/−^ mice may have also amplified antitumor immune responses. In addition to the beneficial effect on GGR, the observed increase in UVB-induced apoptosis and the associated clearance of damaged cells may have also contributed to the reduced SCC development in AHR^−/−^ mice. Indeed, an enhancement of epidermal apoptosis, for instance by topical application of caffeine or resveratrol, has been shown to reduce UVB-induced skin carcinogenesis in mice [34, 35]. Thus, an enhanced stimulation of both defense mechanisms, GGR and apoptosis, is most probably responsible for the reduced SCC occurrence in AHR^−/−^ mice.

**Fig. 6:**
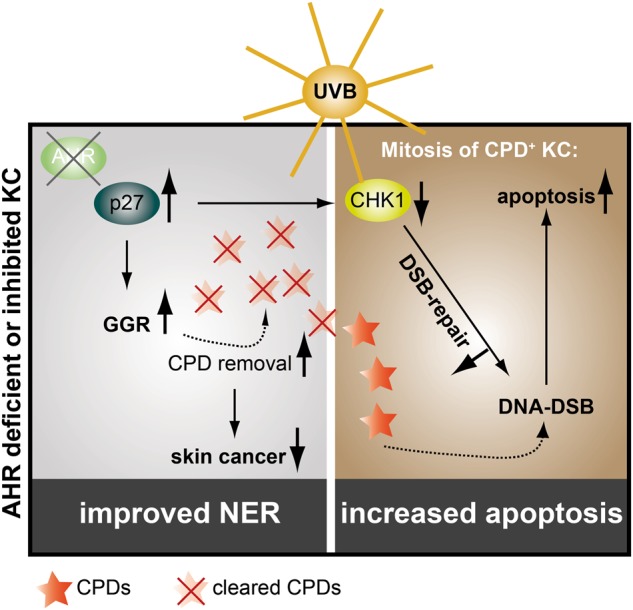
Hypothetical model: AHR inhibition increases p27 protein content resulting in an acceleration of GGR and reduction of mutagenic CPDs and associated SCC development. The increased p27 level results in a reduced CHK1 expression and an attenuation of HRR. Accordingly, CPD-positive AHR-compromised KC are sensitive towards mitosis-related DSBs and subsequent apoptotic cell death

Our results are consistent with the view that an upregulation of the cutaneous p27 protein is causative for the increased GGR in AHR-compromised KC (Fig. 6). The underlying molecular mechanism is quite enigmatic and seems to be independent from p27′s capability to inhibit CDK2/CDK4 and induce cell-cycle arrest. Interestingly, an inhibition of CDK7 seemed to mimic the positive effect of p27 overexpression on NER. CDK7 is the active subunit of the CDK-activating complex, which is part of the multifaceted transcription factor TFIIH. It has been shown that upon UV exposure the CDK-activating complex dissociates from the TFIIH core complex [36], which then switches its function from transcription factor to NER factor [20, 37]. In fact, chemical inhibition of CDK7 has been shown to specifically increase GGR activity [20], thus making it a likely candidate being involved in the stimulation of this repair pathway in AHR-compromised KC. However, in accordance with a previous report [38], we were not able to show a direct protein–protein interaction between CDK7 and p27 in our cell system, indicating that both proteins either interact indirectly via an additional yet to be identified factor or affect GGR through independent mechanisms.

The p27 gene (CDKN1B) is haplo-insufficient for tumor suppression [39, 40], indicating that low p27 levels correlate with cancer proneness. Hence, downregulation of p27 by oncogenic signal transduction occurs frequently in various solid cancers [41], including SCC [42]. Although an effect on p27 transcription was observed in extracutaneous tissues and cells [43], we propose that AHR affects p27 post-translationally, i.e., by activating signal transduction pathways that phosphorylate p27 and target it to the proteasome. The precise underlying mechanism has not yet been identified, however our data clearly point to an involvement of EGFR and downstream PI3K/AKT and MEK/ERK signal transduction. Importantly, AKT [44, 45] and ERK [46, 47] are known to phosphorylate p27 and thereby affect its sub-cellular localization and associated proteasomal degradation. As indicated by our IF stainings, the latter process may involve a CDK2-mediated phosphorylation of p27 at T-187 [22]. Our hypothesis is further supported by previous reports from others and us, showing that the UVB-activated AHR stimulates EGFR and downstream MEK/ERK and PI3K/AKT signaling [10, 48]. A role of EGFR in p27 regulation is also underscored by clinical studies reporting elevated cutaneous p27 protein levels in cancer patients under systemic EGFR inhibitor therapy [49].

As reported earlier [14], AHR inhibition did not reduce but rather enhance UVB-induced apoptosis in HaCaT KC (Fig. 6). The increase of p27 protein upon AHR antagonism resulted in a decreased expression of CHK1, which is required for the initiation of DNA damage responses, i.e., cell-cycle arrest [24] and HRR [25]. Inhibition of CHK1 was shown to enhance UVB-induced KC apoptosis and to prevent photocarcinogenesis [34]. In fact, we have previously observed reduced CHK1 protein levels not only in AHR-compromised human KC, but also in the skin of AHR^−/−^ mice [14]. When UVB-irradiated KC enter mitosis, remaining CPDs may cause a collapse of the replication fork leading to the formation of DSBs [50, 51]. It is therefore highly likely that AHR-compromised KC, due to reduced CHK1 levels, do not properly repair these potent apoptosis-inducing DNA lesions [4], resulting in elevated cell death at later time points (Fig. 6). Accordingly, exposure of CHO cells to dioxin has been shown to accelerate HRR in an AHR-dependent manner [52]. In addition, we have previously reported that AHR is required for proper repair of DSBs induced by ionizing radiation [53] and that reconstitution of CHK1 expression neutralizes the enhanced susceptibility of AHR-silenced KC towards UVB-induced apoptosis [14]. Accordingly, the increased DSB formation observed in AHR-deficient KC is most probably due to an attenuated HRR. Thus, AHR inhibition prevents photocarcinogenesis by accelerating GGR as well as by promoting lethal DSBs in remaining CPD-positive KC (Fig. 6).

Overexpression of AHRR increased p27 protein level, CPD removal and apoptosis, implying that AHRR not only attenuates AHR/xenobiotic-responsive element-dependent responses but also AHR-triggered protein kinase activities. In fact, we have recently reported that the dioxin-induced nuclear accumulation and DNA-binding of C/EBPβ and NF-κB, which is mediated via non-canonical AHR signaling, is reduced in tissues of AHRR Tg mice [21]. However, as AHRR abrogates the growth and malignancy of various human cancers [8], a further elucidation of the link between AHRR and p27 may help to better understand AHRR’s tumor suppressive properties.

In summary, we provide evidence that AHR represses GGR and apoptosis in UVB-exposed KC and critically contributes to skin photocarcinogenesis. The translational relevance of these findings is highlighted by a recent two-stage genome-wide association study identifying AHR as a novel susceptibility locus for SCC in humans [54]. As we have previously shown that AHR antagonism in human skin in vivo is feasible [55], AHR may be a suitable target for topical chemoprevention of UVB-induced skin malignancies.

## Materials and methods

### Cell culture, UVB irradiation and treatment

HaCaT KC were provided by P. Boukamp (DKFZ/IUF) and authenticated by the German Collection of Microorganisms and Cell Cultures (Braunschweig, Germany). The cultivation of HaCaT KC and the generation and cultivation of HaCaT-EV and HaCaT-shAHR KC has been previously described [10]. The source for UVB irradiation was a TL20W/12RS lamp (Philips, Eindhoven, The Netherlands), which emits most of its energy in the UVB range (290–320 nm) with an emission peak at 310 nm. For both UVB and sham exposure, culture medium was replaced by PBS. For cell treatment, Wortmannin, PD153035, PD98059, MG-132, BaP (all from Sigma-Aldrich, Munich, Germany), roscovitine (Enzo Life Sciences, Loerrach, Germany), BS-181 (Selleckchem, Houston, TX, USA), SU9516 (Tocris Bioscience, Bristol, UK) and MNF (provided by I. Meyer, Symrise AG, Holzminden, Germany) were dissolved in DMSO. EGF (Sigma-Aldrich) and Ac-DEVD-CHO (Enzo Life Sciences) were dissolved in water.

### Southwestern slot–blot analyses

Equal amounts of isolated DNA were diluted in TE_10/1_-buffer (pH 8), incubated for 5 min in boiling water and cooled down for 2 min on ice. Samples were spotted on positively charged nitrocellulose membrane (GE Healthcare, Little Chalfont, UK) using a slot–lot chamber coupled to a vacuum manifold. The membrane-bound DNA was denatured for 45 min on Whatmann paper soaked with 0.4 N NaOH. Membranes were blocked overnight in 5% skim milk in TBS-Tween-20 (0.5%; TBS-T) at 4 °C. Membranes were incubated for 2 h at 4 °C with a HRP-conjugated thymine dimer antibody (Kamiya Biomedical Company, Tukwila, WA, USA) in 5% skim milk/TBS-T. Membranes were washed and signals were detected using the WesternBright ECL substrate (Advansta, Menlo Park, CA, USA). The signal intensity of the irradiated control sample was defined as 100%. A serial dilution of this sample was spotted and stained for CPDs to ensure linearity of the signal intensity (example shown in Supplementary Figure S5). The SWB-based CPD detection in sham-exposed KC did not produce any detectable signals and therefore is not shown.

### SDS-PAGE and western blot analyses

Protein isolation, SDS-PAGE and western blot analyses were carried out as described previously [14]. Primary antibodies used in this study were: p27, CDK2, γH2AX, β-actin, GAPDH, STAT3, pSTAT3 Y-705 (all from Cell Signaling Technology, Dancers, MA, USA), XPA (Sigma-Aldrich), α-tubulin (ExBio, Vestec, Czech Republic), AHRR (Novoprotein Scientific, Summit, NJ, USA), AHR, pp27 T-187, pCDK2 T-160, XPC and CSB (all from Santa Cruz Biotechnology).

### Immunohistochemistry

Hematoxylin and eosin (H&E) staining and immunohistochemistry of skin tumors were performed on paraformaldehyde-fixed 7-µm paraffin sections. H&E stainings were mounted in Vectashield medium (Biozol, Eching, Germany). For immunohistochemical staining of tissues, paraffin sections were deparaffinized. Antigens were retrieved by boiling in pH 6 citrate buffer for 12 min. After washing, endogenous mouse Ig's were blocked using M.O.M Blocking Reagent (Biozol) according to the manufacturer´s protocol. Primary antibody (mutant p53, clone PAb 240, Thermo Scientific, Dreieich, Germany) was applied overnight at 4°C in a humidified chamber. Next day, staining was visualized using a peroxidase-based detection kit (Vector AEC Substrate Kit, Biozol) before mounting in gelatine.

### Immunofluorescence staining of cells

KC were grown on collagen I-coated coverslips until subconfluency and fixed with either ice-cold MeOH or 70% EtOH for 10 min at −20 °C. EtOH-fixed cells were permeabilized with 0.1% Triton/PBS for 10 min at RT. Cells were blocked with 5% BSA for 1 h at RT and subsequently incubated with primary antibodies (AHR, p27, pp27 T-187, pCDK2 T-160) diluted in blocking solution. Next day, cells were incubated with AlexaFluor 488- and 568-conjugated secondary antibodies and DAPI for 1 h at RT. Antibodies were diluted in blocking solution and immunostained cells were mounted in Mowiol (Sigma-Aldrich).

### Quantiative real-time PCR

RNA isolation, cDNA synthesis, quantitative real-time PCR and primer sequences were described previously [14].

### Transient RNA interference

Transient transfection of HaCaT KC with XPA, XPC, CSB, p27 and non-silencing siRNA (all from Santa Cruz Biotechnology) was done using INTERFERin reagent (Polyplus Transfection, Illkirch, France).

### Overexpression experiments

Transient transfection of HaCaT KC with pCMV5p27 (provided by J. Massagué, Addgene plasmid #14049 [56]), pcDNA5-rAHRR (provided by Y. Inouye [57]), and respective empty vectors was done using JetPEI reagent (Polyplus Transfection). Efficiency of rat AHRR overexpression was assessed by semi-quantitative PCR.

### Apoptosis assay

Apoptosis was determined by using the Annexin V-FITC Apoptosis kit (BioVision, Mountain View, CA, USA) and a FACSCalibur II device (BD Biosciences, San Jose, CA, USA). In addition, caspase-3 activity was measured by using the Caspase-3 Fluorometric Assay Kit (PromoCell, Heidelberg, Germany) according to the manufacturer’s instructions.

### Neutral comet assay

HaCaT KC were detached with trypsin/EDTA and 20 µl of the cell suspension (approx. 1000 cells) plus 120 µl of 0.5% low melting agarose solution were pipetted on pre-coated superfrost slides, sealed with a cover slip and incubated for 5 min on ice. Slides were transferred to vertical staining jars containing lysis buffer (pH 9.5; 2.5 M NaCl, 100 mM EDTA, 10 mM Tris, 10% DMSO, 1% Triton X-100). After 1 h, slides were washed 3-times with dH_2_O. Next, slides were placed in electrophoresis buffer (pH 8.5; 300 mM NaOH, 1 mM, EDTA) for 20 min followed by 10 min electrophoresis. Slides were washed 3-times with ice-cold neutralization buffer (pH 7.5; 0.4 M Tris), kept in absolute ethanol for 5 min and then air-dried. Slides were stained with ethidium bromide and sealed with cover slips. Fifty cells per treatment were analyzed using a camera-connected microscope (Olympus BX60). Tail moment was used as parameter to characterize the extent of DNA damage.

### Animals and chronic UVB irradiation

Generation, breeding and genotyping of AHR^−/−^ and AHR^+/+^ SKH-1 hairless mice was described previously [14]. The generation, breeding and characterization of transgenic AHRR B6 (AHRR Tg) mice is described in Ref. [21]. All animals were housed in our specific pathogen-free animal facility. For the photocarcinogenesis study, 8 AHR^−/−^ SKH-1 mice and 10 AHR^+/+^ littermates were irradiated over a period of 24 weeks. Sham-exposed mice of each genotype were used as control groups: In week 1 the animals were irradiated 3-times with 90 mJ/cm^2^ UVB, followed by one irradiation-free week. From week 3 on, the mice were irradiated 3-times/week with an initial dose of 60 mJ/cm^2^ UVB, followed by a weekly increase of 10 mJ/cm^2^ until a maximum dose of 150 mJ/cm^2^ UVB was reached (week 12). Mice were further irradiated with this dose until the end of week 24.2 weeks later, animals were sacrificed and tumor numbers were assessed. Tumor samples were embedded in paraffin and the resulting slices were H&E stained and number-coded for blinded histopathological evaluation. Animal experiments were performed according to the national animal care guidelines.

### Acute UVB exposure of mice

For CPD analyses, AHR^+/+^ and AHR^−/−^ SKH-1 mice (except ctrl. animals) were exposed to a single dose of 185 mJ/cm^2^ UVB, and 30 min and 48 h later mice were sacrificed and the dorsal skin of each animal was prepared. Total DNA was isolated from 4 mm punch biopsies using a standard phenol/chloroform extraction protocol.

### Quantification of CPD by HPLC/MS-MS

The HPLC-MS/MS-based detection of CPD in DNA isolated from UVB-irradiated murine skin was performed as described previously [18].

### Statistical analyses

All data shown are mean (± standard deviation) from three or more independent experiments, if not indicated otherwise. In some cases, representative results are shown. Differences were considered significant at *p* ≤ 0.05. A comparison of two groups was made with an unpaired, two-tailed Student’s *T*-test. A comparison of multiple groups was made with analysis of variance followed by a Sidak’s multiple comparison test.

## Electronic supplementary material

Supplementary data
