# Supplementary material for: The AHR represses nucleotide excision repair and apoptosis and contributes to UV-induced skin carcinogenesis
Source: Cell Death Differ. 2018 Jul 16;25(10):1823–36. doi: 10.1038/s41418-018-0160-1 (PMC6180092; doi:10.1038/s41418-018-0160-1)
Supplement: Supplementary file 1 — Supplementary data [file 41418_2018_160_MOESM1_ESM.docx]

**Supplementary Information - Pollet et al.**

**
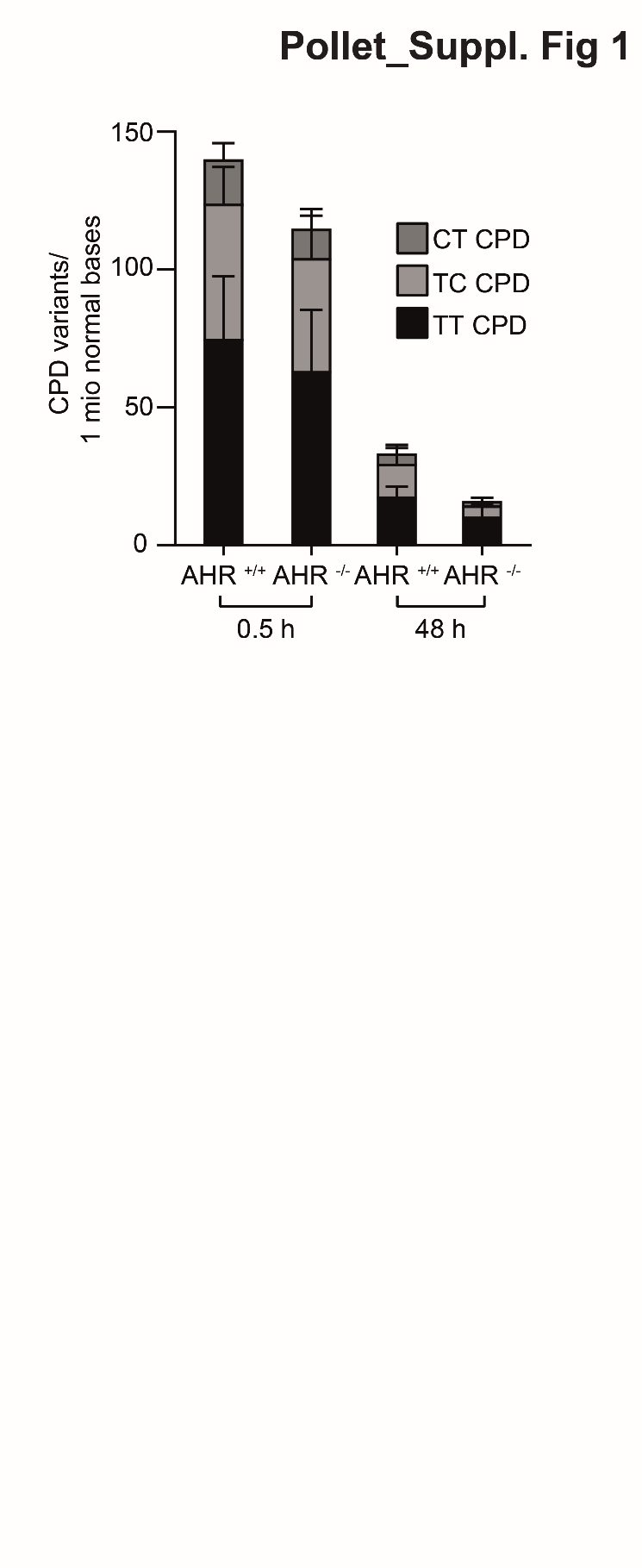
**

**Figure S1.** Distribution between TT, TC and CT CPDs in the skin of UVB-exposed *A*HR^+/+^ and AHR^-/-^ mice. HPLC-MS/MS-based analysis of CPD subtypes in the DNA of skin samples from AHR^+/+^ and AHR^-/-^ SKH-1 mice 0.5 h and 48 h after exposure to a single dose of 185 mJ/cm^2^ UVB.

**
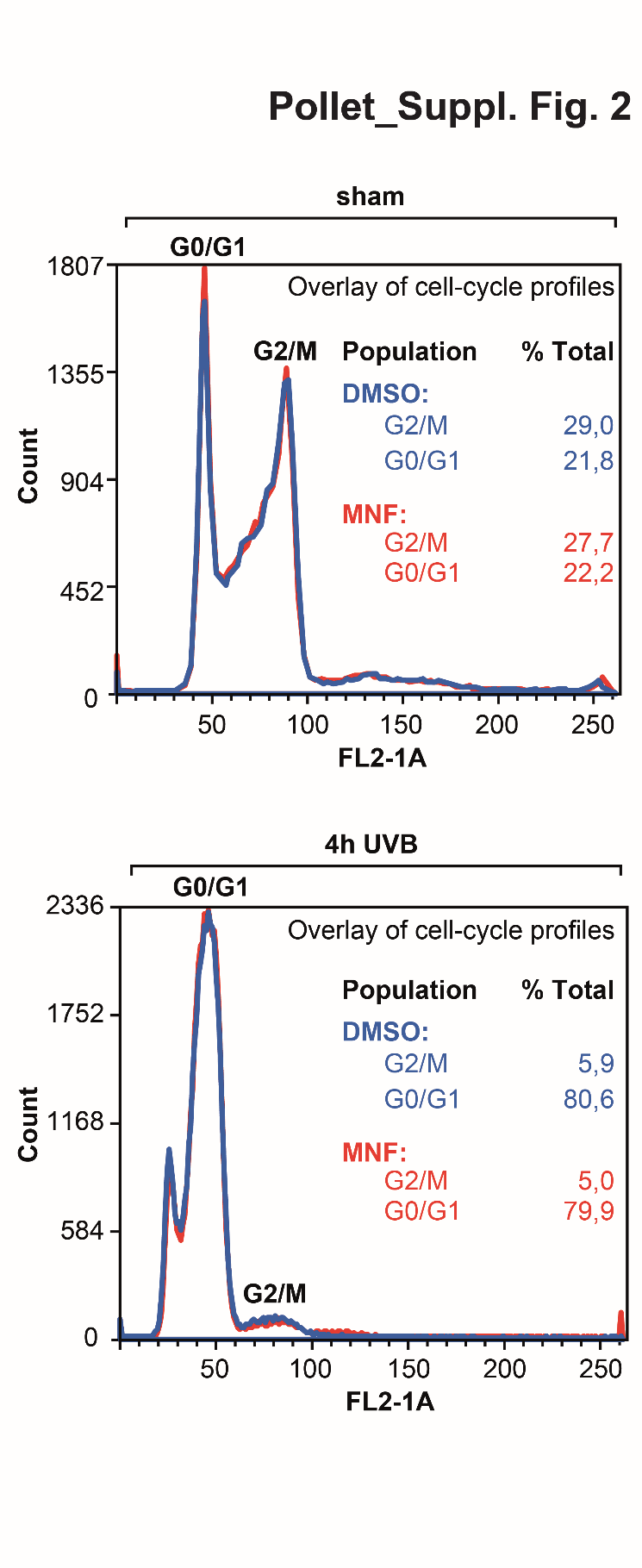
**

**Figure S2.** Merged cell-cycle profiles of sham and UVB-irradiated (200 J/m^2^) HaCaT KC treated with 0.1% DMSO or 20 µM MNF. Cells were harvested 4 h after sham/UVB irradiation and prepared for FACS analysis.

**
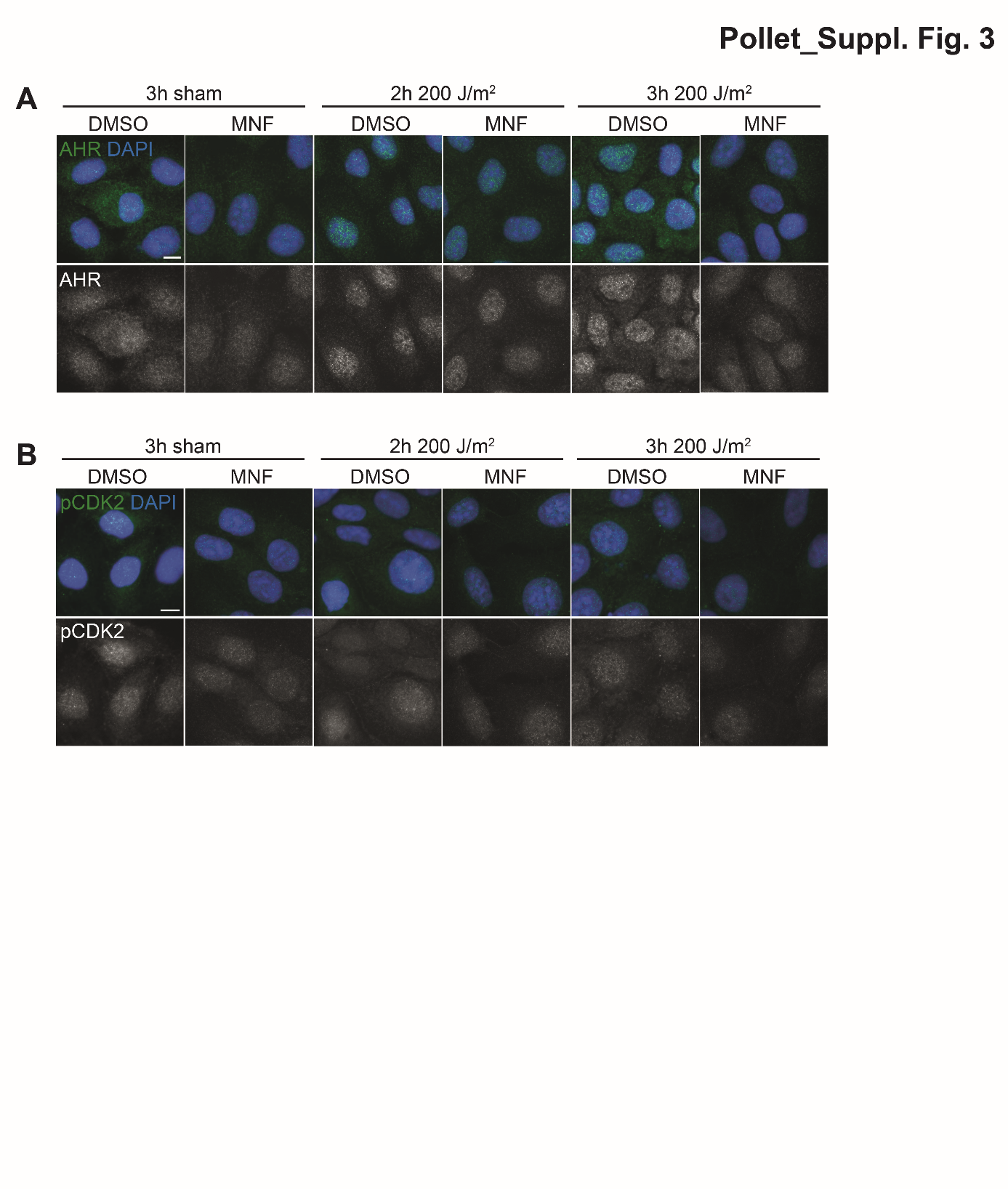
**

**Figure S3.** Effect of AHR antagonism on the expression and localization of AHR and pCDK2 in sham- and UVB-irradiated HaCaT KC. HaCaT KC were irradiated with 0 and 200 J/m^2^ UVB and treated with 20 µM or 0.1% DMSO. After 2 h and 3 h, cells were fixed and antibody stained for **(A)** AHR and **(B)** pCDK2 T-160 (scale: 10 µm).

**
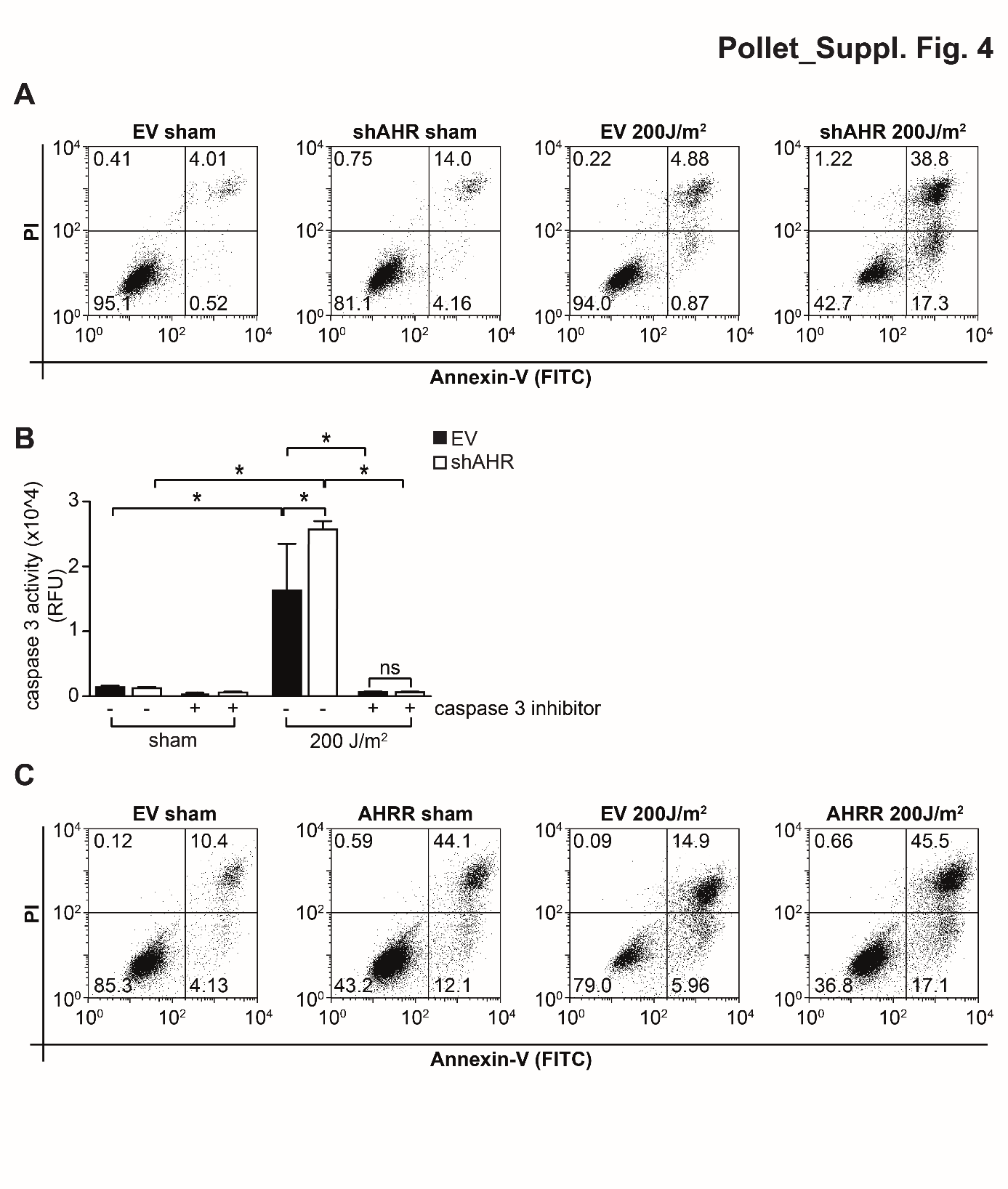
**

**Figure S4.** AHR-compromised KC exhibit an enhanced susceptibility to UVB-induced apoptosis. **(A)** Representative FACS plots of the Annexin V / propidium iodide staining of sham- and UVB-irradiated HaCaT-EV and HaCaT-shAHR cells. **(B)** HaCaT-EV and HaCaT-shAHR KC were irradiated with 200 J/m^2^ UVB and subsequently treated with 20 µM Ac-DEVD-CHO or solvent. After 24 h, caspase-3 activity was assessed as described under Material and Methods. **(C)** Representative FACS plots of the Annexin V / propidium iodide staining of sham- and UVB-irradiated control and AHRR-overexpressing HaCaT KC.

**
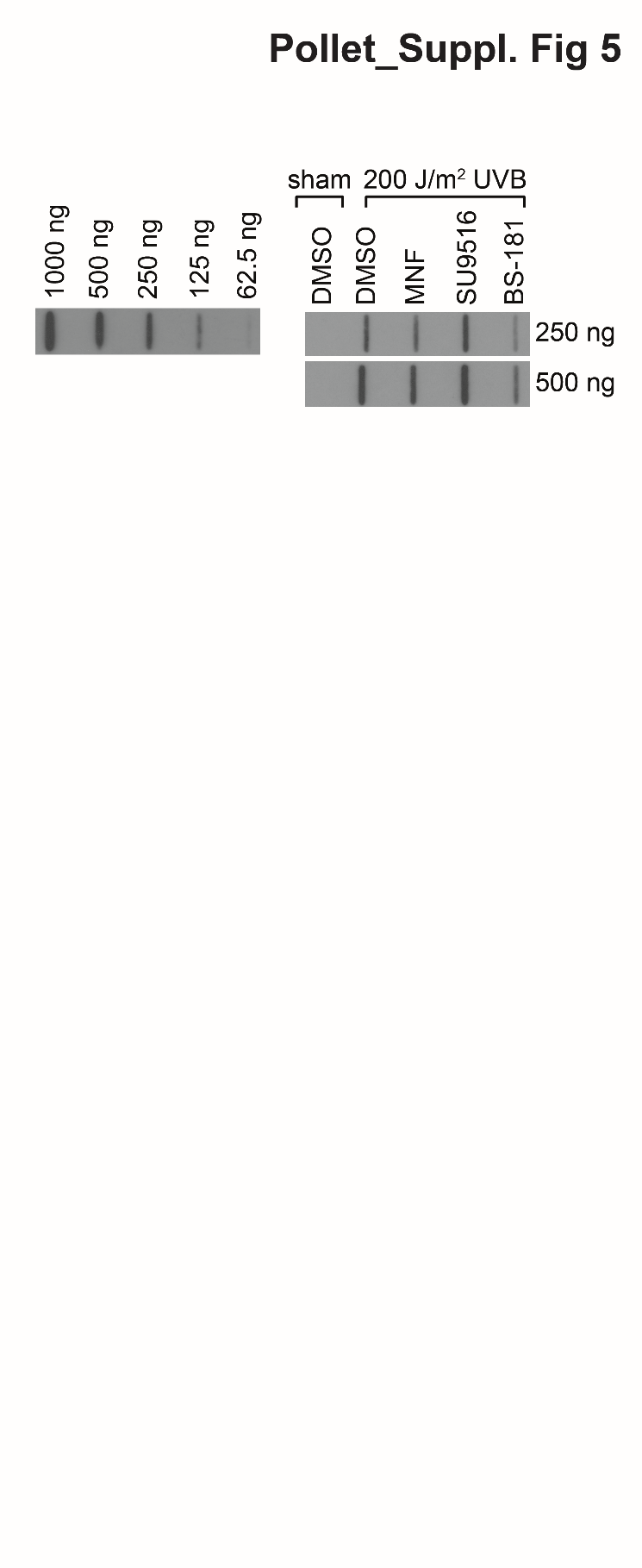
**

**Figure S5.** Representative SWB analysis of UVB-induced CPDs. On the left, a serial dilution of the sample expected to contain the highest amount of CPDs (here: DNA from DMSO-treated cells isolated 4 h after exposure to 200 J/m^2^ UVB) is shown. On the right, different DNA amounts (250 ng and 500 ng) from all samples of the experiment (here: influence of two CDK inhibitors and MNF on CPD content 4 h after exposure to 200 J/m^2^ UVB) were spotted on nitrocellulose and stained for CPDs.
